# Patient-Centered Attitudes and Associated Factors Among Medical Students in Spain: A Cross-Sectional Study

**DOI:** 10.7759/cureus.112842

**Published:** 2026-07-17

**Authors:** Lourdes V Quiles-Sánchez, Georgios Kyriakos, Juan A Sánchez-Sánchez, Miriam Moñino-García, Juan F Menárguez-Puche

**Affiliations:** 1 Roldán Primary Care Health Centre, Servicio Murciano de Salud, Murcia, ESP; 2 Department of Endocrinology and Nutrition, Hospital General Universitario Santa Lucía, Cartagena, ESP; 3 Department of Social and Health Sciences, Faculty of Medicine, University of Murcia, Murcia, ESP; 4 Department of Public Health Sciences, Faculty of Medicine, University of Murcia, Murcia, ESP; 5 Molina-Jesús Marín Primary Health Centre, Servicio Murciano de Salud, Murcia, ESP

**Keywords:** interpersonal and communication skills, medical students, patient-centered care, ppos, psychometric tests, shared decision-making, spain

## Abstract

Introduction: Patient-centered care (PCC) is fundamental to healthcare quality, yet no data on patient-centered attitudes among medical students in Spain exist. This study assessed these attitudes using the Patient-Practitioner Orientation Scale (PPOS), identified associated factors, and compared findings internationally.

Methods: A cross-sectional survey was conducted of all six year groups at the University of Murcia, Spain, using the 18-item PPOS administered online. Bivariate and multivariate analyses were performed.

Results: Of 1,300 students invited, 359 participated (27.6% response; 66.3% female). Scores indicated patient-centered orientations (total PPOS 4.41 ± 0.40; Caring 4.82 ± 0.45; Sharing 4.01 ± 0.53). Female students scored higher across all scales (p < 0.001; d = 0.51). Sharing scores were the lowest dimension across all subgroups, regardless of sex, year, or motivation. No statistically significant difference was observed between preclinical and clinical students after Bonferroni correction, though this comparison remains inconclusive given the Caring subscale's limited reliability (α = 0.50) and unequal participation across year groups. In multiple linear regression, female sex and being in a relationship were significant predictors; complementary logistic regression confirmed these associations (OR = 0.44 and 0.58, respectively), though overall explained variance was low (R² < 0.08).

Conclusions: Spanish medical students showed patient-centered orientations compared with international data. The consistently low Sharing scores suggest that students endorse empathic care more readily than shared decision-making, identifying the latter as a priority for communication skills training. The Caring subscale's limited reliability points to the need for a psychometrically validated Spanish version.

## Introduction

Patient-centered care (PCC), defined as care that is respectful of and responsive to individual patient preferences, needs, and values [1,2], is central to high-quality healthcare delivery. PCC has been associated with improved patient satisfaction, better clinical outcomes, and reduced healthcare costs [3,4].

Assessing PCC attitudes during medical education is important, as the values and beliefs that students develop during training shape their future communication behaviors in clinical practice [5]. A central question is whether clinical exposure weakens or reinforces patient-centered attitudes. Several studies have reported a decline in PCC attitudes as students progress through clinical years [6,7], attributed in part to implicit institutional norms and clinical pressures [8,9]. However, this trajectory is not universal: when institutional cultures actively promote compassion and effective communication, clinical exposure can sustain or even strengthen patient-centered attitudes [10]. A second consistent finding is that female students hold more patient-centered attitudes than their male peers [11-14].

The Patient-Practitioner Orientation Scale (PPOS), developed by Krupat et al. [15], is the most widely used instrument to measure PCC attitudes among healthcare students and professionals. This 18-item scale differentiates between patient-centered and doctor-centered orientations across two dimensions: Sharing (willingness to share information, power, and control with patients) and Caring (attention to patients' emotions, expectations, and psychosocial context). The PPOS has been translated into at least eight languages, although psychometric inconsistencies in factor structure and internal consistency across translations have been noted, particularly for the Caring subscale [16]. To our knowledge, no study has examined the psychometric properties of the Spanish translation in a medical student population.

A meta-analysis pooling data from 20 studies found a mean PPOS score of 4.16 (95% CI: 3.95-4.37) among healthcare students globally [11]. Cross-cultural studies have revealed substantial variation, with Western European and American students tending to score higher than those in East Asian and South Asian settings [11,17-19].

Despite this growing evidence base, PCC attitudes among medical students in Spain have not been studied. The only Spanish PPOS data come from Perestelo-Pérez et al. [20], who reported a mean PPOS of 4.46 (SD 0.73) in 321 primary care professionals. No comparable data exist for students. Spain's universal public healthcare system is built on a strong primary care foundation, which may expose students to patient-centered role models from early clinical contact [21]. The progressive feminization of the medical profession in Spain (women now represent approximately 65% of medical students) could also influence PCC attitudes. Understanding these contextual factors is relevant for Spanish curricular development and for international comparisons within the broader Southern European context.

This study had three aims: to describe PCC attitudes among medical students at the University of Murcia using the PPOS, establishing the first baseline for this population; to analyze the association of sociodemographic, academic, and motivational factors with PPOS scores; and, as an exploratory objective, to examine whether PCC attitudes differed between preclinical and clinical training stages.

## Materials and methods

Study design and reporting

This was a cross-sectional study conducted at the Faculty of Medicine, University of Murcia, Spain, between February and May 2019. The study adhered to the Strengthening the Reporting of Observational Studies in Epidemiology (STROBE) guidelines for cross-sectional studies [22]; the completed checklist is provided as Appendix 1.

Setting

The Faculty of Medicine at the University of Murcia offers an undergraduate-entry, six-year medical degree. The first three years (1st-3rd) comprise predominantly preclinical coursework, while the latter three years (4th-6th) include extensive clinical rotations in hospital and primary care settings. At the time of data collection, approximately 1,300 students were enrolled across all six year groups.

Participants and data collection

All enrolled medical students were invited to participate via an anonymous, self-administered online questionnaire distributed through the university's virtual learning platform (encuestas.um.es). The survey link was sent by email to all enrolled students, with periodic reminders during the data collection period (February-May 2019). Because the study aimed to survey all enrolled students (census approach), no a priori sample size calculation for a specific effect size was performed. Cognitive pre-testing of the questionnaire was conducted with a convenience sample of sixth-year students (n = 21) in December 2018 to ensure clarity and comprehension. No additional cultural adaptation or pilot validation beyond cognitive pre-testing was performed prior to the main data collection.

Instrument

The PPOS uses a six-point Likert response format. After reverse coding of relevant items, scores range from one (most doctor-centered) to six (most patient-centered) [15]. The scale yields a total score and two subscale scores: Sharing (nine items) and Caring (nine items). The Spanish translation of the PPOS was accessed through the University of Miami El Centro Measures Library [23]. Permission to use the PPOS in the present study was granted by Professor Edward Krupat, developer and copyright holder of the instrument (personal communication, June 2026). To date, to our knowledge, no study has examined the psychometric properties of the Spanish PPOS translation in a medical student population. The internal consistency data reported here represent a preliminary contribution toward this goal.

In addition to the PPOS, the questionnaire collected sociodemographic, academic, and motivational variables. Sociodemographic variables included sex, age, nationality, religious beliefs, relationship status, type of school attended (public vs. private/subsidized), number of languages spoken, and university entrance examination score. Academic variables included year of study, preferred future specialty (laboratory-based, medical-surgical, surgical, or clinical), and participation in research or extracurricular activities. Motivational variables captured the primary reason for choosing medicine (humanitarian, social prestige, economic, power/influence, or family influence). The questionnaire also included 11 additional items derived from prior focus group research with students at the same institution; these items are not analyzed in the present report.

Statistical analysis

Data were analyzed using IBM SPSS Statistics for Windows, Version 24 (Released 2016; IBM Corp., Armonk, New York, United States). Internal consistency was assessed with Cronbach's alpha for the total PPOS and both subscales; item-level analyses (item-total correlations and alpha-if-item-deleted) were conducted for the Caring subscale, given its anticipated low reliability [16,20]. Descriptive statistics included means, standard deviations, and 95% confidence intervals for continuous variables and frequencies with percentages for categorical variables.

For bivariate analyses, several variables were dichotomized based on theoretical relevance and sample distribution: religious beliefs (religious (Christian, Islam, Buddhism, and other; n = 186) vs. atheist/agnostic (n = 173)), relationship status (in a relationship/married vs. single/other), and year of study (preclinical (1st-3rd) vs. clinical (4th-6th)). Bivariate comparisons of PPOS scores were performed using independent-samples t-tests (or Mann-Whitney U tests when normality was not confirmed by Shapiro-Wilk tests) for dichotomous variables and one-way analysis of variance (ANOVA) (or Kruskal-Wallis) with Bonferroni-corrected post-hoc pairwise comparisons for polytomous variables. Effect sizes were calculated as Cohen's d. Given six primary bivariate comparisons, a Bonferroni-adjusted significance threshold of p < 0.008 was applied alongside the conventional p < 0.05 threshold.

Multiple linear regression models were constructed with total PPOS, Sharing, and Caring as dependent variables. Separate models were fitted for each category of predictors (sociodemographic, academic, and motivational), given the exploratory nature of the study; within each category, variables that reached significance (p < 0.05) in bivariate analyses were entered as predictors. Assumptions were verified for multicollinearity (tolerance > 0.1), autocorrelation (Durbin-Watson), residual normality (Shapiro-Wilk), and homoscedasticity (visual inspection of residual plots). Only complete cases were analyzed; item-level missing data were negligible (<1%), and no imputation was performed. As a complementary analysis, binary logistic regression was also performed to provide clinically interpretable odds ratios; the rationale and methodology are described alongside the results.

Ethical considerations

The study protocol was reviewed and approved by the Ethics Committee of the Faculty of Medicine, University of Murcia (no formal reference number was assigned for this anonymized survey study). All procedures were performed in accordance with the Declaration of Helsinki. Informed consent was obtained from all participants, who voluntarily completed the anonymous online questionnaire. No identifiable personal data was collected.

## Results

Participant characteristics

**Figure 1:**
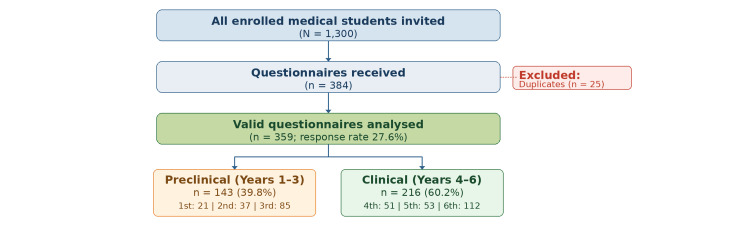
Participant flow diagram (n = 359) Students were classified as preclinical (years 1-3; n = 143, 39.8%) or clinical (years 4-6; n = 216, 60.2%).

Of the 1,300 students invited, 384 responded to the survey. After excluding 25 entries identified as duplicates based on identical response timestamps and content, 359 valid questionnaires were analyzed, yielding an overall response rate of 27.6% (Figure 1). 

Participation varied substantially across year-groups (range: n = 21 for first-year to n = 112 for sixth-year). Students in clinical years (4th-6th) comprised 60.2% (n = 216) of the sample. The sex distribution (66.3% female) was consistent with the overall composition of the faculty. The demographic characteristics of participants are summarized in Table 1. 

**Table 1:** Participant characteristics (n = 359) Values are n (%) unless otherwise stated.

Characteristic	n (%) or mean ± SD
Sex	
Female	238 (66.3)
Male	121 (33.7)
Age (years), mean ± SD	22.97 ± 4.75
Training stage	
Preclinical (years 1–3)	143 (39.8)
Clinical (years 4–6)	216 (60.2)
Year of study	
1st	21 (5.8)
2nd	37 (10.3)
3rd	85 (23.7)
4th	51 (14.2)
5th	53 (14.8)
6th	112 (31.2)
Nationality	
Spanish	348 (96.9)
Other	11 (3.1)
Religious beliefs	
Religious	186 (51.8)
Atheist/agnostic	173 (48.2)
Relationship status	
In relationship/married	154 (42.9)
Single/other	205 (57.1)
Type of school	
Public	240 (66.9)
Private/subsidized	119 (33.1)
Primary motivation for medicine	
Humanitarian	296 (82.5)
Economic	22 (6.1)
Social prestige	17 (4.7)
Family influence	13 (3.6)
Power/influence	11 (3.1)

PPOS scores and internal consistency

**Figure 2:**
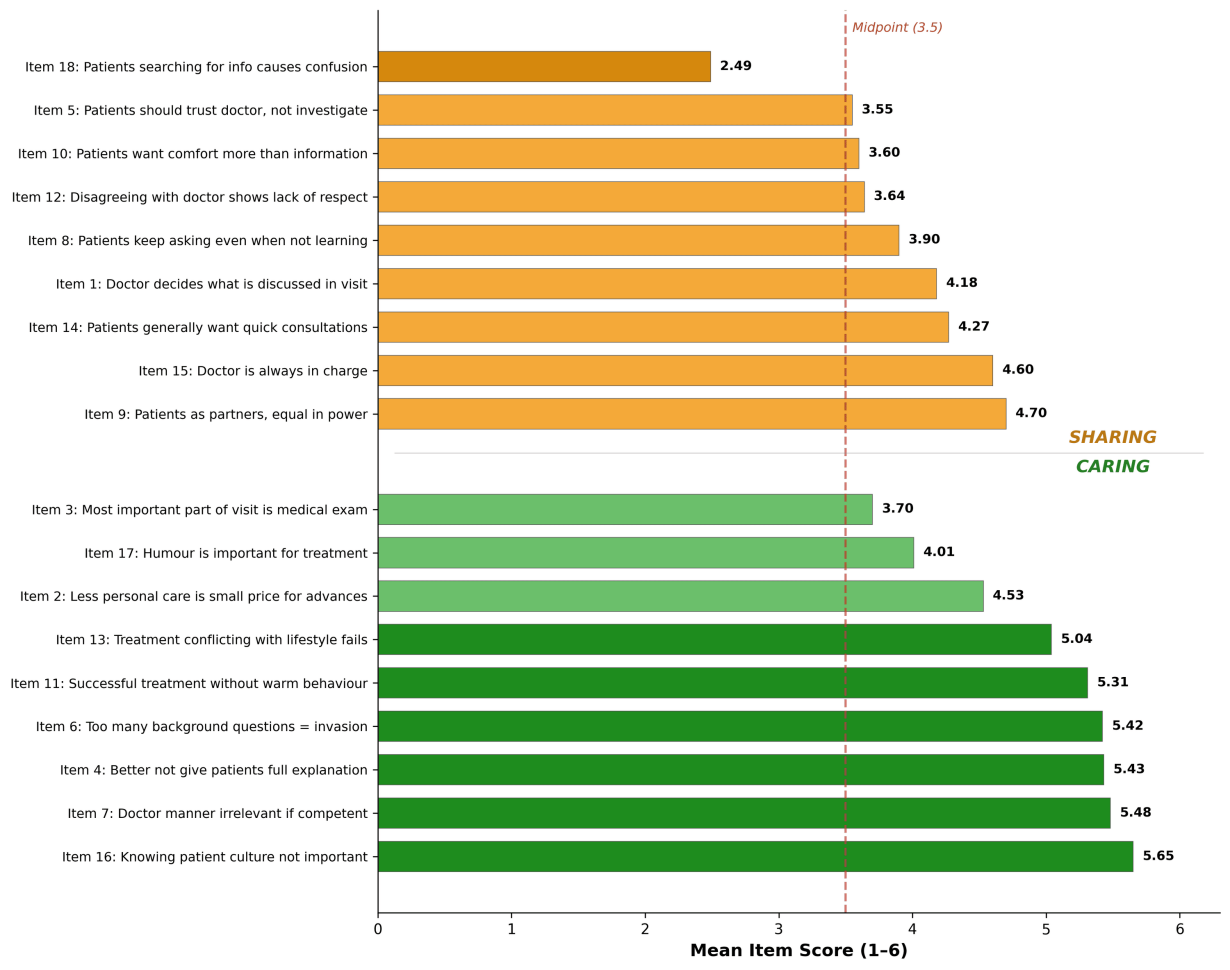
Item-level PPOS scores (n = 359) Horizontal bars represent mean scores for each of the 18 PPOS items, grouped by subscale (orange = Sharing; green = Caring). Items are sorted by score within each subscale. The dashed red line indicates the scale midpoint (3.5). Higher scores reflect more patient-centered attitudes. PPOS: Patient-Practitioner Orientation Scale

The mean total PPOS score was 4.41 (SD 0.40; 95% CI: 4.37-4.45), above the scale midpoint of 3.5. Subscale means were 4.82 (SD 0.45; 95% CI: 4.77-4.87) for Caring and 4.01 (SD 0.53; 95% CI: 3.95-4.06) for Sharing. Cronbach’s alpha was 0.67 for the total PPOS, 0.63 for the Sharing subscale, and 0.50 for the Caring subscale. Given this low internal consistency, findings pertaining to the Caring dimension should be regarded as exploratory. Exploratory item-deletion analysis showed that no single item removal improved the Caring α above 0.55. Item-level scores across both subscales are shown in Figure 2. 

Bivariate analyses

**Figure 3:**
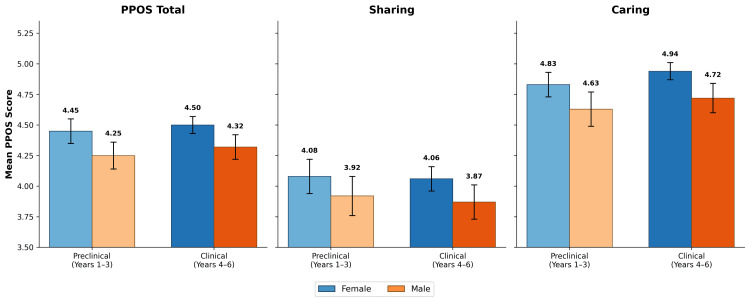
PPOS scores by sex and training stage (n = 359) Preclinical female n = 96; preclinical male n = 47; clinical female n = 142; clinical male n = 74. Lighter bars = preclinical (years 1-3); darker bars = clinical (years 4-6). Error bars represent 95% confidence intervals. PPOS: Patient-Practitioner Orientation Scale

Table 2 summarizes the key bivariate findings. Female students scored significantly higher than males on the total PPOS (4.50 vs. 4.25; p < 0.001; d = 0.51), the Sharing subscale (4.07 vs. 3.92; p = 0.008; d = 0.30), and the Caring subscale (4.93 vs. 4.63; p < 0.001; d = 0.52). These sex differences remained significant after Bonferroni correction (adjusted threshold p < 0.008). The pattern of PPOS scores by sex and training stage is illustrated in Figure 3.

**Table 2:** Mean PPOS scores by sex and training stage (n = 359) Shapiro-Wilk tests confirmed adequate normality for all comparisons; independent-samples t-tests were used throughout. Values are mean (95% CI). Standard deviations: total PPOS = 0.40; Sharing = 0.53; Caring = 0.45 (overall sample). p₁ = total PPOS; p₂ = Sharing; p₃ = Caring. d = Cohen's d (d for total PPOS sex difference = 0.51; d for Caring sex difference = 0.52). *Survives Bonferroni correction (adjusted threshold p < 0.008). †Religious includes Christian (n = 180), Islam (n = 3), Buddhism (n = 1), and other (n = 2). Single/other includes single (n = 201) and separated/divorced (n = 4). PPOS: Patient-Practitioner Orientation Scale

Variable	n	PPOS total mean (95% CI)	t	p₁	Sharing mean (95% CI)	t	p₂	Caring mean (95% CI)	t	p₃	d
Sex											
Female	238	4.50 (4.45–4.55)	4.56	<0.001*	4.07 (4.00–4.14)	2.68	0.008*	4.93 (4.87–4.99)	4.03	<0.001*	0.51
Male	121	4.25 (4.17–4.33)			3.92 (3.82–4.02)			4.63 (4.53–4.73)			
Training stage											
Preclinical (1st–3rd)	143	4.37 (4.30–4.44)	1.76	0.080	3.99 (3.89–4.09)	0.46	0.644	4.75 (4.67–4.83)	2.50	0.013	0.27
Clinical (4th–6th)	216	4.44 (4.39–4.49)			4.02 (3.95–4.09)			4.87 (4.81–4.93)			
Primary motivation											
Humanitarian	296	4.43 (4.38–4.48)	2.19	0.029	4.03 (3.97–4.09)	1.45	0.148	4.84 (4.79–4.89)	1.78	0.076	0.27
Non-humanitarian	63	4.33 (4.22–4.44)			3.93 (3.78–4.08)			4.73 (4.60–4.86)			
Relationship status											
In a relationship	154	4.45 (4.39–4.51)	1.98	0.048	4.05 (3.96–4.14)	1.22	0.224	4.86 (4.79–4.93)	1.28	0.202	0.22
Single/other†	205	4.38 (4.32–4.44)			3.97 (3.90–4.04)			4.79 (4.73–4.85)			
Religious beliefs											
Religious†	186	4.41 (4.35–4.47)	0.12	0.908	4.01 (3.93–4.09)	0.02	0.982	4.81 (4.74–4.88)	0.25	0.801	0.02
Atheist/agnostic	173	4.41 (4.35–4.47)			4.01 (3.93–4.09)			4.83 (4.76–4.90)			
School type											
Public	253	4.40 (4.35–4.45)	0.48	0.629	4.00 (3.94–4.06)	0.29	0.770	4.80 (4.74–4.86)	0.88	0.380	0.06
Private/subsidized	106	4.42 (4.34–4.50)			4.02 (3.91–4.13)			4.85 (4.76–4.94)			

Students in a relationship or married scored higher on the total PPOS (4.46 vs. 4.38; p = 0.043; d = 0.20), though this did not survive Bonferroni correction. Atheist or agnostic students scored higher on the Caring subscale than religious students (4.88 vs. 4.77; p = 0.018; d = 0.25); this difference also did not survive Bonferroni correction. Students reporting humanitarian motivation scored higher across all dimensions than those citing other motivations (total PPOS: 4.44 vs. 4.28; p = 0.009; d = 0.40); this effect approached but did not reach the Bonferroni-adjusted threshold.

Regarding academic variables, clinical-year students (4th-6th) scored higher on the Caring subscale than preclinical students (4.87 vs. 4.75; p = 0.023; d = 0.27), while no significant differences were observed for the total PPOS (p = 0.255) or the Sharing subscale (p = 0.775). This Caring difference did not survive Bonferroni correction (p > 0.008) and should be interpreted with caution, given the low reliability of the Caring subscale (α = 0.50) and the unequal representation across year-groups (range: n = 21 to n = 112). No significant differences were found for nationality, type of school attended, number of languages spoken, or university entrance examination score.

Multivariate analyses

Separate multiple linear regression models were fitted for sociodemographic, academic, and motivational predictors. In the sociodemographic model for total PPOS, female sex (B = 0.219; p < 0.001) and being in a relationship (B = 0.105; p = 0.013) were significant predictors; religious beliefs approached significance (B = 0.081; p = 0.053). This model explained 7.7% of variance (adjusted R² = 0.077; F = 8.44; p < 0.001). In the motivational model, humanitarian motivation (B = 0.175; p = 0.002) and social prestige motivation (B = -0.159; p = 0.008) were both significant predictors, explaining 4.0% of variance (adjusted R² = 0.040). Academic variables (training stage, preferred specialty) did not significantly predict total PPOS scores (F = 1.81; p = 0.165). The proportion of variance explained by the examined variables was small across all models, indicating that additional unmeasured factors contribute substantially to PCC attitudes.

As a complementary analysis, binary logistic regression was performed to provide clinically interpretable odds ratios (Table 3). The total PPOS was dichotomized at the mean (≥ 4.42 = high vs. < 4.42 = low), as the variable was normally distributed (Shapiro-Wilk p = 0.125). Male sex was associated with lower odds of high PPOS scores (OR = 0.44; 95% CI: 0.28-0.70; p < 0.001), and being single was associated with lower odds relative to being in a relationship (OR = 0.58; 95% CI: 0.37-0.90; p = 0.016). Neither age nor religious beliefs reached significance in the adjusted model. The model's Nagelkerke R² was 0.066, with a sensitivity of 64.5% and a specificity of 51.5%, reflecting the limited discriminative capacity inherent to dichotomizing continuous data. These results should be interpreted as confirmatory of the linear regression findings rather than as a primary analysis; the low R² is consistent with the small effect sizes observed in the linear models and with the broader PPOS literature, where sociodemographic variables typically explain under 10% of attitudinal variance.

**Table 3:** Binary logistic regression for total PPOS (n = 359; sociodemographic predictors) Dependent variable: Total PPOS dichotomized at the sample mean (≥4.42 = high patient-centered orientation), as the variable was normally distributed (Shapiro-Wilk p = 0.125). ᵃ Reference category. Model Nagelkerke R² = 0.066. Hosmer-Lemeshow goodness-of-fit: p > 0.05. Model sensitivity = 64.5%; specificity = 51.5%, estimated using the classification table at the default 0.50 probability cutoff. This analysis is presented as complementary to the linear regression models to provide clinically interpretable odds ratios. PPOS: Patient-Practitioner Orientation Scale; OR: odds ratio; CI: confidence interval; SE: standard error

Variable	Category	B	SE	Wald	OR	95% CI	p
Sex	Male ᵃ				1.00		
	Female	-0.82	0.24	11.98	0.44	0.28–0.70	<0.001
Age	≤20 years ᵃ				1.00		
	21–23 years	-0.15	0.28	0.28	0.86	0.50–1.49	0.597
	≥24 years	-0.36	0.33	1.17	0.70	0.36–1.34	0.279
Relationship status	Single/other ᵃ				1.00		
	In a relationship	-0.55	0.23	5.88	0.58	0.37–0.90	0.016
Nationality	Non-Spanish ᵃ				1.00		
	Spanish	-0.39	0.55	0.51	0.68	0.23–1.98	0.475
Religious beliefs	Atheist/agnostic ᵃ				1.00		
	Religious	0.05	0.22	0.05	1.05	0.68–1.63	0.822
School type	Private/subsidized ᵃ				1.00		
	Public	0.12	0.24	0.24	1.12	0.70–1.80	0.626
Training stage	Preclinical ᵃ				1.00		
	Clinical	-0.28	0.23	1.48	0.76	0.48–1.19	0.224
Motivation	Non-humanitarian ᵃ				1.00		
	Humanitarian	-0.35	0.29	1.44	0.71	0.40–1.25	0.231

## Discussion

This is, to our knowledge, the first study to assess PCC attitudes among medical students in Spain using the PPOS. The principal findings were: Spanish medical students scored above the global pooled mean and comparably to Spanish primary care professionals; female sex was the strongest and most consistent predictor of patient-centered attitudes; no statistically significant difference between preclinical and clinical students was observed after Bonferroni correction, though this comparison remains inconclusive given psychometric and sampling limitations; and the PPOS showed suboptimal internal consistency, consistent with psychometric concerns raised in the international literature.

Overall PCC orientation in the international context

**Figure 4:**
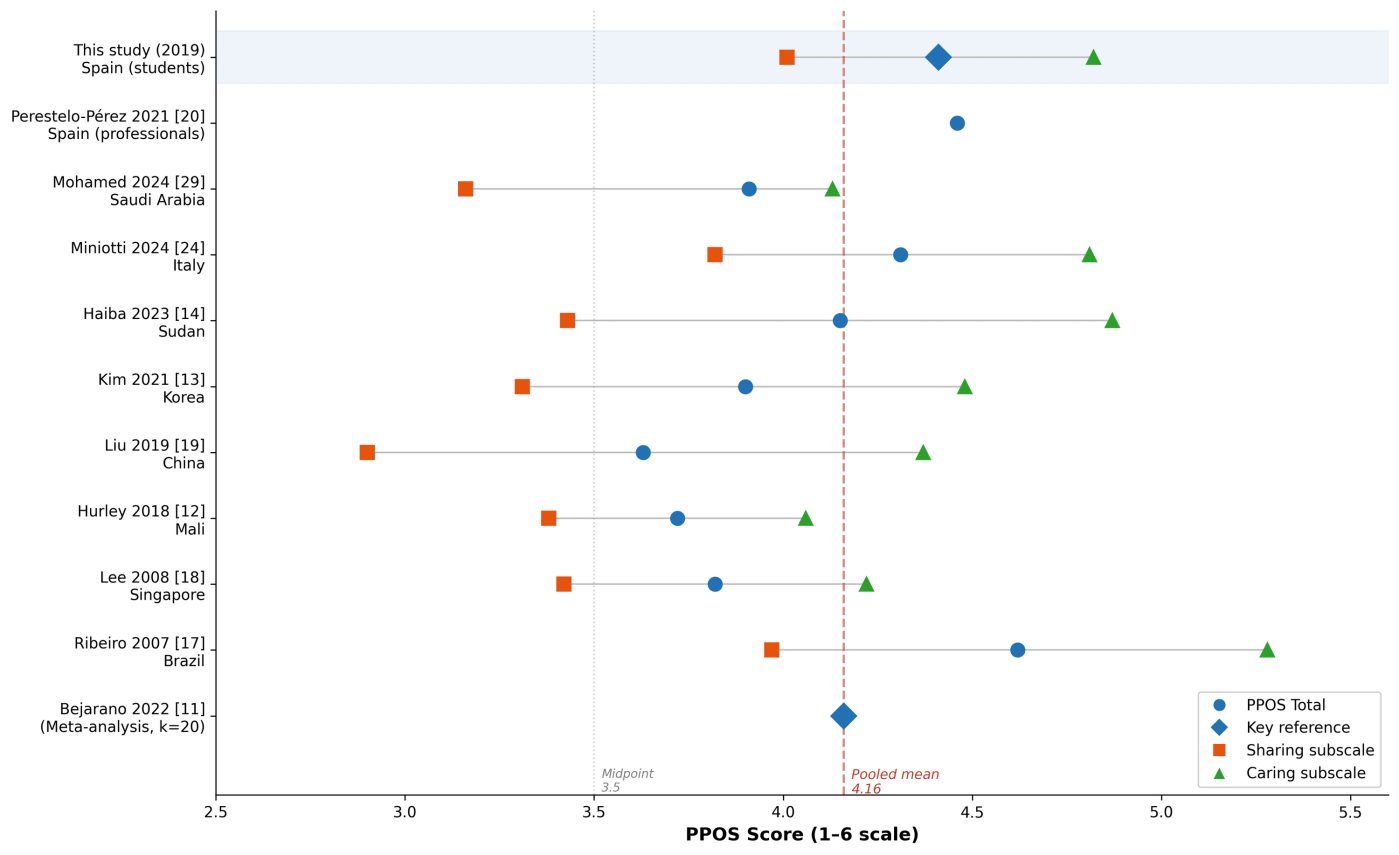
International comparison of PPOS scores Circles = PPOS Total; squares = Sharing subscale; triangles = Caring subscale. Diamond markers denote key references (pooled meta-analytic mean and present study). The dashed red line indicates the pooled global mean (4.16) from Bejarano et al. [11]. The grey dotted line indicates the scale midpoint (3.5). Grey lines connect subscale scores within each study. The present study is highlighted in blue. PPOS: Patient-Practitioner Orientation Scale

The mean total PPOS score of 4.41 exceeds the pooled global estimate of 4.16 (95% CI: 3.95-4.37) reported in the Bejarano et al. meta-analysis [11] and is comparable to the 4.46 reported among Spanish primary care professionals (Figure 4) [20]. Spanish medical students thus appear to hold moderately patient-centered attitudes, broadly in the range of other Western European settings. However, direct comparison of raw PPOS scores across countries assumes measurement invariance, which has not been established for most translations [16,24]. Pauli and Wilhelmy [25] demonstrated measurement invariance for a short six-item German version (PPOS-D6), but the invariance status of the full 18-item Spanish version remains unknown.

As in virtually every cultural setting where the PPOS has been used [11,18,20,26], Caring subscale scores (4.82) were substantially higher than Sharing scores (4.01). Part of this gap may reflect social desirability: Caring items assess attitudes towards holistic, empathic care that are broadly socially valued, whereas Sharing items challenge the traditional medical hierarchy by asking about power-sharing and information disclosure [15,27]. This Caring-Sharing gap is not unique to our sample; it has been reported in Brazil, China, and Sudan, and suggests that medical students across cultural settings find it easier to endorse empathic care than to accept patients as equal partners in clinical decisions. The practical relevance is that training focused on empathy alone may leave the more resistant attitudes towards power-sharing unaddressed. The item-level score profile confirms this heterogeneity, with items 18 (information-seeking) and five (trusting the doctor) scoring below the midpoint, identifying specific areas where students held more doctor-centered views.

Clinical-year students and the Caring subscale

In this sample, no statistically significant difference between preclinical and clinical students was observed after Bonferroni correction. The uncorrected Caring subscale comparison (4.87 vs. 4.75; p = 0.023; d = 0.27) should be regarded as exploratory and inconclusive rather than as evidence of attitudinal stability across training stages.

This finding must be interpreted with several important caveats. First, this is a cross-sectional comparison between different student cohorts, not a longitudinal measurement of the same students over time. The observed difference could reflect cohort effects (e.g., different admission characteristics across years), differential attrition, or curricular changes between year groups. Second, the finding did not survive Bonferroni correction (adjusted threshold p < 0.008). Third, the Caring subscale's low internal consistency (α = 0.50) introduces substantial measurement error, which attenuates observed effect sizes: the true difference between groups could be larger or smaller than d = 0.27, and the direction of the bias is indeterminate. Fourth, participation was markedly unequal across year groups (range: n = 21 for first-year to n = 112 for sixth-year), with clinical-year students comprising 60.2% of the sample versus the expected 50% under equal enrollment. This imbalance means that the preclinical subsample may be less representative of its population than the clinical subsample, and neither weighting nor sensitivity analyses were feasible given the absence of population-level data on non-responders.

Nevertheless, similar stability has been reported in settings where institutional cultures promote compassion and communication [10,14,28]. Ardenghi et al. [28] found in a longitudinal Italian study that Sharing improved over time while Caring remained stable. The Spanish healthcare system's strong primary care orientation [21] may expose students to patient-centered role models during clinical rotations, potentially counteracting the decline observed in more hospital-centric settings. However, any inference about the protective role of institutional features remains speculative given the cross-sectional design.

Sex as the most consistent predictor

Female sex was the most consistent predictor of patient-centered attitudes, reaching significance across all PPOS dimensions in both bivariate and multivariate analyses (d = 0.51). In the complementary logistic regression, male sex was associated with lower odds of high PPOS scores (OR = 0.44; 95% CI: 0.28-0.70). This is unsurprising: the Bejarano et al. meta-analysis [11] confirmed a pooled sex effect favoring women, and virtually every PPOS study, from China [19] to Sudan [14], Korea [13], Italy [24,28], and Saudi Arabia [29], reports higher scores among females.

With women comprising 66.3% of our sample, communication skills training should target all students, particularly the Sharing dimension. Whether the observed sex difference reflects socialization, response style, or genuine attitudinal divergence remains unclear and would require mixed-methods research.

Other associated factors

Being in a relationship was associated with higher total PPOS scores in both linear (B = 0.105; p = 0.013) and complementary logistic regression (OR = 0.58; p = 0.013; d = 0.20), though this finding requires replication given its small effect size. Humanitarian motivation predicted higher PCC scores (B = 0.175; p = 0.002), while social prestige motivation was associated with lower scores (B = -0.159; p = 0.008), consistent with previous studies [15,19]. Atheist students scored higher on Caring (p = 0.018), but given the subscale's low reliability, this should be considered exploratory.

Psychometric considerations

The internal consistency values observed in this study (total α = 0.67; Sharing α = 0.63; Caring α = 0.50) fall below the conventional 0.70 threshold but are comparable to those reported by Krupat et al. [15], Lee et al. [18], and the only other Spanish PPOS study (α Caring = 0.55) [20]. Rather than being unique to our sample, these values appear to reflect a broader problem with the scale's structural validity. The low Caring reliability may be partly attributable to cultural factors: in Southern European and Latin American contexts, where empathic and relational dimensions of care are deeply embedded in social norms, the Caring items may lack discriminative power because respondents endorse them uniformly, producing restricted variance and reduced internal consistency.

Miniotti et al. [24] demonstrated in 1,543 Italian medical students that the PPOS exhibits significant multidimensionality inadequately captured by the original two-factor model, particularly affecting the Caring subscale. A study [27] proposed a four-factor structure with superior model fit, and another [16] confirmed in their scoping review that factor structure varies across translations. A psychometrically validated Spanish PPOS version, based on exploratory and confirmatory factor analysis in Spanish-speaking samples, is clearly needed, potentially following a shortened adaptation such as the PPOS-D6 approach [25], to improve reliability and enable meaningful cross-cultural comparisons. An important limitation of the present study is that no pilot exploratory factor analysis was conducted prior to the main data collection; future studies using the Spanish PPOS should incorporate such preliminary psychometric work.

Strengths and limitations

The main strengths of this study are that it provides the first PPOS data from Spanish medical students, that it includes motivational predictors rarely examined in PPOS studies, and that data were collected in 2019, prior to the COVID-19 pandemic, which provides a baseline for future comparisons.

Several limitations must be acknowledged. First, this is a cross-sectional study from a single institution; findings cannot be generalized to other Spanish medical schools without replication, and as noted above, the cross-sectional design precludes causal inference regarding training stage. Second, the response rate of 27.6% introduces potential selection bias; students with stronger PCC attitudes may have been more likely to participate, which could inflate scores. No data on non-responders were available, precluding formal non-response analysis. However, several indirect indicators suggest that the sample was not severely biased: the sex distribution (66.3% female) was consistent with the overall faculty composition and with national figures for Spanish medical schools (≈65%); the mean age (22.97 ± 4.75 years) was consistent with expected values for a six-year program; and participants were present across all six year groups. Nonetheless, participation varied substantially by year (estimated response rates ranging from approximately 10% in the first year to 52% in the sixth year, assuming roughly equal enrollment across year groups), with clinical-year students comprising 60.2% of the sample versus the expected 50% under equal participation. This differential participation means that the preclinical-clinical comparison should be interpreted with particular caution: the clinical-year subsample may be more representative of its population than the preclinical subsample, introducing an asymmetry that could bias between-stage comparisons in either direction.

Third, the PPOS is a self-report instrument susceptible to social desirability bias, particularly for the Caring items. Fourth, the regression models explained less than 8% of variance, indicating that unmeasured factors, empathy, personality, and specific educational experiences are likely more important determinants of PCC attitudes. Fifth, these data were collected in 2019, seven years prior to the present publication. During this interval, the COVID-19 pandemic fundamentally altered medical education delivery in Spain, with prolonged periods of remote learning, reduced clinical contact, and significant psychological strain on students and faculty. Additionally, Spain's primary care system experienced substantial workforce pressures during and after the pandemic, which may have affected the clinical learning environment and the role models available to students. Post-pandemic evidence suggests that empathy decline during clinical training persists [30], and pre- vs. post-pandemic comparisons remain scarce. The present 2019 dataset thus constitutes a pre-pandemic baseline whose current applicability should not be assumed; replication with post-pandemic cohorts is essential to determine whether these findings remain valid.

Finally, the construct of "patient-centeredness" continues to evolve [26], and the PPOS may not capture all its dimensions. Of note, the two principal findings, the sex effect and the low explained variance, were observed on the total PPOS and Sharing subscales (α = 0.67 and 0.63, respectively); the low Caring reliability primarily affects the preclinical-clinical comparison, which we have framed as exploratory and inconclusive throughout.

## Conclusions

To our knowledge, this is the first study using the PPOS among Spanish medical students and found generally patient-centered orientations that compare favorably with international data. Female sex was the most consistently associated factor, but the low overall explained variance makes it clear that PCC attitudes are shaped by factors largely beyond the sociodemographic and academic variables examined here. No statistically significant difference between preclinical and clinical students was observed after correction for multiple comparisons; this comparison remains inconclusive given the Caring subscale's limited reliability, the unequal participation across year groups, and the cross-sectional design.

The consistently low Sharing scores across all subgroups identify shared decision-making as a concrete target for communication skills training; empathy-focused modules alone may not address students' resistance to sharing power and information with patients. Efforts to promote patient-centered attitudes should focus on what institutions do rather than on who their students are.

These pre-pandemic data, collected seven years before publication, provide a historical baseline whose current applicability should not be assumed. Three priorities emerge for future research: psychometric validation of the Spanish PPOS through exploratory and confirmatory factor analysis, replication with post-pandemic cohorts to assess whether these findings remain valid, and prospective studies examining whether communication skills training translates into changes in PCC attitudes and clinical practice.

## Appendices

Appendix 1 

**Table 4:** STROBE Statement (checklist for cross-sectional studies) STROBE: Strengthening the Reporting of Observational Studies in Epidemiology [22]

Item No.	STROBE Recommendation	Reported	Location in manuscript
	Title and abstract		
1	(a) Indicate the study's design with a commonly used term in the title or the abstract	Cross-sectional study	Title, Abstract
	(b) Provide in the abstract an informative and balanced summary	Structured IMRAD abstract	Abstract
	Introduction		
2	Explain the scientific background and rationale	PCC importance, PPOS instrument, gap in Spanish data	Introduction
3	State specific objectives	Three aims stated	Introduction
	Methods		
4	Present key elements of study design early in the paper	Cross-sectional study, Feb–May 2019	Section 2
5	Describe the setting, locations, and relevant dates	University of Murcia, Faculty of Medicine	Section 2
6	(a) Give the eligibility criteria and selection methods	All enrolled students (census); online questionnaire	Section 2
7	Clearly define all outcomes, exposures, and predictors	PPOS total, Sharing, Caring; sociodemographic, academic, motivational variables	Section 2
8	For each variable, give sources of data and methods of assessment	PPOS 18-item scale; Spanish translation from U. of Miami	Section 2
9	Describe any efforts to address potential sources of bias	Anonymous questionnaire; cognitive pre-testing; duplicate removal	Section 2
10	Explain how the study size was arrived at	Census approach; no a priori sample size calculation	Section 2
11	Explain how quantitative variables were handled	Dichotomisation criteria described; Bonferroni correction	Section 2
12	(a) Describe all statistical methods	t-tests, ANOVA, linear regression, logistic regression	Section 2
	(b) Describe methods to examine subgroups and interactions	Preclinical vs. clinical; descriptive subgroup comparison by sex and training stage (Figure 2)	Sections 2, 3
	(c) Explain how missing data were addressed	Complete case analysis; <1% item-level missing; no imputation	Section 2
	(d) If applicable, describe analytical methods for the sampling strategy	Not applicable (census)	N/A
	(e) Describe any sensitivity analyses	Bonferroni correction; item-deletion analysis for Caring	Sections 2, 3
	Results		
13	(a) Report numbers of individuals at each stage	Figure 1: 1,300 invited → 384 responded → 359 valid	Section 3; Figure 1
	(b) Give reasons for non-participation at each stage	25 duplicates excluded; reasons for non-response unknown	Section 3
	(c) Consider the use of a flow diagram	Figure 1 provided	Section 3
14	(a) Give characteristics of study participants	Table 1: demographics of 359 participants	Section 3; Table 1
	(b) Indicate the number with missing data for each variable	<1% item-level missing	Section 2
15	Report numbers of outcome events or summary measures	Mean PPOS scores ± SD with 95% CIs	Section 3
16	(a) Give unadjusted and confounder-adjusted estimates	Table 2 (bivariate); Table 3 (logistic regression)	Section 3
	(b) Report category boundaries when variables were categorized	Mean (4.42) for PPOS total; median (4.78) for Caring	Section 2
17	Report other analyses done	Linear regression by predictor category; Bonferroni thresholds	Section 3
	Discussion		
18	Summarise key results with reference to study objectives	Opening paragraph with 4 principal findings	Section 4
19	Discuss the limitations of the study	Cross-sectional design, 27.6% response, α=0.50, R²<8%, pre-pandemic, social desirability	Section 4
20	Give a cautious overall interpretation	International context, exploratory Caring findings, psychometric concerns	Section 4
21	Discuss the generalisability of the study results	Single institution; replication needed	Section 4
22	Give the source of funding and the role of the funders	No specific funding	Funding
